# Optimal positioning of right-sided internal jugular venous catheters: Comparison of intra-atrial electrocardiography *versus* Peres' formula

**DOI:** 10.4103/0972-5229.40943

**Published:** 2008

**Authors:** Anish M. Joshi, Guruprasad P. Bhosale, Geeta P. Parikh, Veena R. Shah

**Affiliations:** **From:** Department of Anesthesia and Critical Care, GR Doshi and KM Mehta Institute of Kidney Diseases and Research Centre, Dr. HL Trivedi Institute of Transplantation Sciences, Civil Hospital campus, Asarwa, Ahmedabad - 380 016, Gujarat, India

**Keywords:** Central venous catheterization, intra-atrial electrocardiography, Peres' height formula

## Abstract

Central venous catheters are routinely placed in patients undergoing major surgeries where expected volume and hemodynamic disturbances are likely consequences. The incorrect positioning may give false central venous pressure (CVP) readings leading to incorrect volume replacement and other serious complications. 50 American Society of Anaesthesiologists grade II-IV patients aged 18-60 years were selected for right-sided internal jugular vein (IJV) catheterization using Seldinger's technique. In group A, central venous catheterization was done under electrocardiography (ECG) guidance. In group B, the catheter was inserted blindly using Peres' formula of “height (in cm)/10”. The position of the tip of central venous catheter was confirmed radiologically by postoperative chest X-ray. 92% of patients in group A had radiologically correct positioning of catheter tip i.e. above the carina, while in group B 48% patients had over-insertion of the catheter in to the right atrium. Intra-atrial ECG technique to judge correct tip positioning is simple and economical. It can determine the exact position intraoperatively and can justify a delayed postoperative chest X-ray to confirm CVC line tip placement.

## Introduction

Central venous catheters (CVC) are routinely placed in patients undergoing major surgeries where expected volume and hemodynamic disturbances are likely consequences, and/or for multiple infusion administration during surgery or later for parenteral nutrition postoperatively. The correct position of the tip of CVC is considered to be in the superior vena cava (SVC) above the level of pericardial reflection. Blood flow conditions are then optimal to keep the catheter away from the intima and to dilute the infused drugs immediately. Anaesthesiologists usually place the lines in the operating room preoperatively and the chest X-ray is done postoperatively to confirm the correct placement several hours later. Because incorrect placement can lead to serious complications such as cardiac tamponade, perforation or dysrhythmias caused by interaction with the vessel wall or the endocardium,[1] it would be desirable to confirm correct positioning after placement in the operating room. The best way to confirm correct position is to perform the procedure under fluoroscopy or to obtain a chest X-ray postprocedure, but it is costly, associated with radiation exposure and would lengthen the operative time.

Various landmarks,[2,3] simple formulae[4,5] and sophisticated techniques like right atrial ECG[6,7] and transesophageal echocardiography[7,8] have been developed to ensure correct placement of the CVC tip. Peres' utilized the patient's height to predict the optimal length of the catheter to be inserted by different approaches and demonstrated that 24% terminated in right atrium.[9] Another technique is to place the CVC under ECG guidance which was first described by Hellerstein and colleagues in 1949[10] and has been claimed to detect the intraatrial position of the tip of CVCs by detecting an intraatrial P-wave (P-atriale) with the exploring electrode.

The aim of this study was to compare the intra-atrial ECG technique with Peres' height formula for optimal placement of CVC and establish it's accuracy by postoperative chest X-ray.

## Materials and Methods

The protocol was approved by the institutional ethics committee and informed consent were obtained from 50 patients aged 18-60 years undergoing elective surgery and scheduled to receive a CVC as part of their anaesthetic management were enrolled in this prospective study. Height of all patients in cms was recorded at the time of a preoperative visit, and the patients were randomly divided into 2 groups of 25 each. Patients with altered coagulation parameters or having cardiac disorders like atrial fibrillation, multifocal ventricular premature beats, supraventricular tachycardia, left bundle branch block or pacemaker were excluded from the study.

In both the groups, after successful puncture of the right internal jugular vein (IJV), a 15/20 cm single lumen CVC (Certofix^®^ Mono 16G, B. Braun Melsungen, Germany) was inserted over 50 cm guidewire using the Seldinger technique. In Group A, the guidewire was withdrawn through the catheter until a mark on the guidewire indicated the tip to be exactly positioned at the tip of the catheter. A connection between the guidewire and the ECG adapter (Certodyn^®^ - Universal adapter, B. Braun Melsungen, Germany, Cost Rs. 9000.00) was established by connecting the reference red electrode to a universal adapter equipped with a switching function on the right thoracic side to record a modified lead II. The yellow electrode was placed on the left shoulder and the neutral green electrode was placed on the lower left chest. By turning the switch of the universal adapter, intra-atrial ECG could be recorded [Figure 1A and 1B].

**Figure 1:**
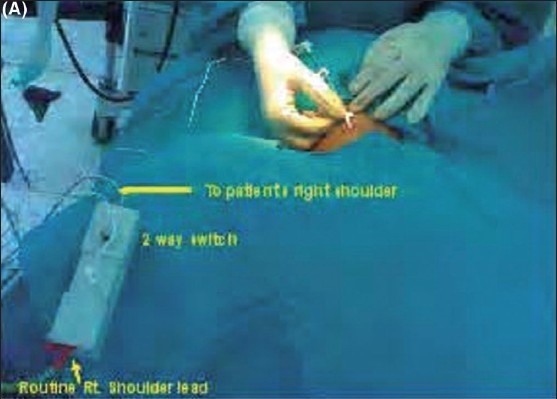
Modified bipolar lead II being recorded by universal adapter (A) Actual

**Figure 1:**
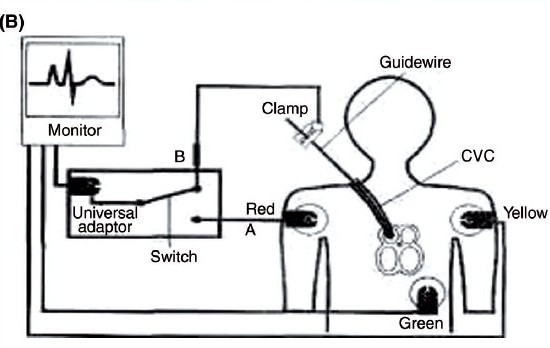
Modified bipolar lead II being recorded by universal adapter (B) Diagrammatic

While advancing the guidewire alongwith the catheter, the configuration of the P wave was seen on the ECG monitor. Gradually the height of the P wave increased and it became equal to or more in amplitude than the R wave [Figure 2A]. On further advancement, the P wave became bifid, but at this point the catheter and guidewire were immediately withdrawn till the P wave became of the normal configuration [Figure 2B]. The CVC was fixed with stitches and sterile dressing was applied.

**Figure 2:**
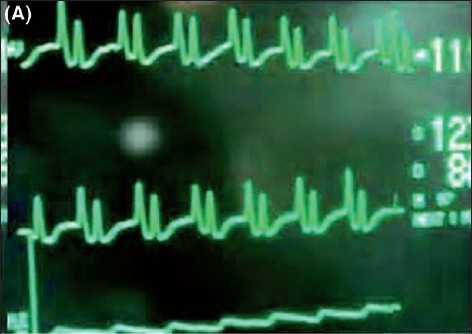
(A) Intraatrial ECG in lead II where P wave is larger than R wave

**Figure 2:**
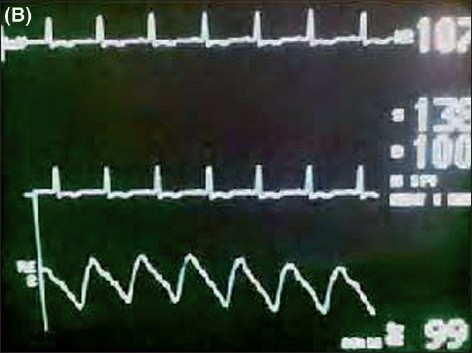
(B) Intraatrial ECG in lead II showing a normal P wave

In Group B, the catheter was blindly inserted and final insertion depth was kept as per the Peres' formula of “height (in cm)/10”.[4] A postoperative portable chest X-ray anteroposterior was done in all cases in supine position for assessing the position of the catheter's tip. Chest X-rays were read by one radiologist who was aware of the study protocol but blinded to the group allocation. CVC position was judged as correct if the tip was positioned above the level of carina and as overinsertion when the tip was below the level of carina. In both the groups, final insertion depth, incidence of arrhythmias during the CVC placement, insertion time and complications (arterial puncture, hematoma, pneumothorax) were recorded.

## Data and Statistical Analysis

Demographic data are presented as mean ± SD. Correct CVC position, incidence of arrhythmias and complications are presented as percentages. Data were analyzed using the unpaired Student's t test. *P* < 0.001 was considered to be highly significant.

## Results

Both groups were comparable with respect to age, body weight, sex and height [Table 1].

**Table 1:** Demographics

**Parameter**	**Group A (n = 25)**	**Group B (n = 25)**
Age (Yrs)	59 ± 1.7	58.6 ± 0.95
Weight (Kg)	55 ± 1.58	55.6 ± 1.27
Height (cms)	157.96 ± 6.67	158.84 ± 8.78
Male/Female	13/12	15/10

The mean insertion time was 6.5 min in Group A as compared to 5 min in Group B. No arrhythmias were noted in Group A, while 3 of 25 patients (12%) had dysrhythmias in Group B on insertion which was corrected immediately by pulling back the catheter by 2 cm. The mean length of the IJV catheter inserted in Group A was 12.24 ± 1.30 cm compared to 15.88 ± 0.88 cm in Group B, the difference of 3.64 cm was highly significant (*P* < 0.001) [Table 2].

**Table 2:** Procedural data

**Parameter**	**Group A (n = 25)**	**Group B (n = 25)**
Insertion time (seconds)	390 ± 56.24	300 ± 47.43
Final length of insertion (in cm)	12.24 ± 1.30*	15.88 ± 0.88*
Complications			
Arrhythmias during the procedure	0(0%)	3(12%)
Arterial puncture	9(36%)	6(24%)	
Haematoma	1(4%)	0(0%)	
Pneumothorax	0(0%)	0(0%)	

Mean ± S.D. *P value < 0.0001 (Unpaired student's t Test)

On postoperative chest X-ray in 23 of 25 (92%) cases in Group A the IJV catheter was properly positioned, while in 12 of 25 cases (48%) there was overinsertion in Group B. Also there was coiled catheter in 6 of 25 cases (24%) in Group B while in 2 of 25 cases (8%) in Group A [Table 3].

**Table 3:** Position of central venous catheter

	**Group A (n = 25)**	**Group B (n = 25)**
Properly positioned	23(92%)	7(28%)
Over insertion	0(0%)	12(48%)
Malpositioned IJV catheter	2(8%)	6(24%)

## Discussion

Depending on the point of entry or indication for a CVC, the optimal site for CVC tip placement varies.[11] However, guidelines for CVC placement recommend that the catheter tip should lie above the pericardial reflection[3,9,11–13] to prevent serious and potentially lethal complications like cardiac tamponade, malignant arrhythmias, placement in coronary sinus and tricuspid valve damage. The upper limit of the pericardial reflection cannot be seen on a plain chest X-ray, but it is generally accepted to be below the carina. This has been assessed in preserved[2] and fresh cadavers,[14] in anesthetized children undergoing cardiac surgery[15] and in adults using computerized tomograms.[16] Moreover, its location is preserved even in pulmonary pathology due to its fixation with connective tissue, parallax effect is limited due to its central location and small sagittal distance between it and SVC and it is easily visible even in a poor quality portable anteroposterior chest X-ray. Hence we considered carina as a radiological landmark for CVC tip position.

Various methods have been suggested to estimate the expected length of CVC at the time of insertion. Pere P.W. studied correlation between the length of catheter inserted and patient's height and observed that catheters inserted through right IJV from midcervical point or lower puncture to Height/10cm ended in SVC, while those inserted more than Height/10 + 1cm, 47% ended in right atrium.[9] In our study, we observed 48% incidence of overinsertion with Height/10cm formula with insertion at cricoid level. This may be because we considered carina as reference point for correct positioning whereas Pere PW.[9] considered SVC/RA junction as optimal tip positioning.

Good quality intra-atrial ECG and clear display of the P-wave on the ECG monitor are essential for successful guidance and positioning of CVCs. Consequently, a limitation of ECG guidance is that it cannot be reliably used in patients with atrial fibrillation or other supraventricular arrhythmias. Analysis of P wave morphology as a marker of ECG-guided central venous catheterization has been evaluated previously and most of these studies report variable results. Jeon *et al.*,[7] undertook a prospective descriptive clinical study using transoesophageal echocardiography (TEE) guidance to establish the absolute locations and ranges of CVC tip positions when specific P wave patterns are displayed during ECG-guided central venous catheterization. They concluded that the tallest peaked P wave may be used to place the CVC tip at the SVC/RA junction, the normally-shaped P wave identifies the mid to upper SVC and a biphasic pattern of the P wave can be used to locate the RA. Based on this, we considered normally-shaped P waves as correct positioning and avoid overinsertion in ECG guided group.

Previous studies have indicated the usefulness of ECG guided positioning of CVC placement.[6,17] In a prospective, controlled, randomized trial comparing ECG guidance to conventional placement, Francis *et al.*,[18] reported a significantly higher success rate (96% v/s 59%) using a continuous column of normal saline for ECG-guided CVC placement. Gebhard RE *et al.*, reported 96% correct positioning in group ECG and 76% in group NO-ECG.[19] In our study, success rate was 92%, however we could not prevent malposition in two patients but identified it as decrease rather than increase in the height on further advancement of the CVC which was confirmed on postoperative chest X-ray. We did not try repositioning maneuvers in these cases but it could have resulted in correction of this misplacement and higher incidence of success. CVC placement in the RA occurred only in group B and was associated with a mean insertion depth of 15.88 ± 0.88 cm. Based on this, we recommend inserting CVCs via the Right internal jugular vein (RIJV) no deeper than Height/10-2 cm if ECG guidance cannot be used.

One of the limitations of this study is that all CVCs were placed through the RIJV which is used more frequently than the left internal jugular vein (LIJV) as the access vessel for CVC placement. Therefore, no conclusion can be drawn whether ECG guidance results in more accurate positioning when either the LIJV or one of the subclavian veins are chosen as access routes to the SVC. Schummer *et al.*,[20] suggested that ECG guidance does not improve the accuracy of the CVC placed through the LIJV as it is not capable of providing information about angle of the CVC tip in relation to the wall of the SVC. However, it is believed that ECG guidance is efficient in positioning CVCs into a major vessel just proximal to the heart.

## Conclusion

Intra-atrial ECG guided placement is a useful tool for accurate positioning of RIJV-CVC. The procedure is reliable compared to height formula and requires universal adaptor which is not expensive. It can avoid radiologic confirmation in uncomplicated insertions minimizing cost and radiologic exposure to patients and health care workers.
